# Editorial: Molecular Mechanisms Underlying Polyamine Functions in Plants

**DOI:** 10.3389/fpls.2017.00014

**Published:** 2017-01-24

**Authors:** Patrick H. Masson, Taku Takahashi, Riccardo Angelini

**Affiliations:** ^1^Laboratory of Genetics, University of Wisconsin-MadisonMadison, WI, USA; ^2^Graduate School of Natural Science and Technology, Okayama UniversityOkayama, Japan; ^3^Laboratory of Biochemistry, Physiology and Biotechnology of Plants, Department of Science, University “Roma Tre”Rome, Italy

**Keywords:** polyamines, putrescine, spermidine, spermine, cadaverine, stress, ROS, GABA

Polyamines are small organic bases that have been found in all living organisms where they play important roles in the regulation of growth, development and responses to biotic and abiotic stimuli. They do so by modulating a variety of cellular processes such as cell division, expansion, differentiation, death, nucleic acids, and protein synthesis and function. This diversity of functions derives in part from the ability of polyamines to interact with negatively charged sites in molecules such as nucleic acids, proteins, and lipids. It also derives from their ability to conjugate with a variety of macromolecules such as phenolic compounds and proteins. Furthermore, some products of polyamine catabolism have been found to play critical signaling roles in a variety of physiological, cellular, and developmental processes.

In plants, polyamines have also been found to regulate growth, development, and responses to biotic and abiotic cues. Therefore, it is no surprise that recent attempts at manipulating the signaling pathways leading to polyamines metabolism and/or function have been successful at improving plant growth, affecting development and/or improving tolerance to environmental stress (Alcázar and Tiburcio, 2014). Yet, despite an abundance of recent research publications discussing polyamines metabolism and function in plants, much remains to be elucidated to allow the development of better strategies aimed at designing plants with improved tolerance to the stresses associated with global warming, generating crops with more effective growth in marginal lands to allow food, feed, and fiber production for a rapidly expanding world population, and increasing the production of biomass with improved characteristics for biofuel production.

This research topic aims at highlighting recent advances in plant polyamine research. We collected 14 manuscripts focusing on the metabolism of polyamines and the roles they play in the regulation of plant growth, development, environmental stress response, and stress mitigation. By inviting authors with a broad range of expertise and demonstrated contributions to the field, we aimed at promoting interactions between research teams, reviewers and editors, and linking together different disciplines to resolve longstanding questions related to polyamines metabolism and function in plants.

Two groups of important polyamines have been found to play important roles in the regulation of plant growth, development, and environmental responses: (1) the better characterized putrescine and its derived polyamines, and (2) the less-characterized cadaverine. Most of the papers included in this topic describe research aimed at characterizing the metabolism of, and functions associated with the first group of compounds.

Putrescine is a diamine that contributes to plant stress response and also serves as a precursor in the biosynthesis of higher-level polyamines such as spermidine, spermine, and thermospermine. The last step of putrescine synthesis in plants is catalyzed by a N-carbamoylputrescine amidohydrolase (CPA), an enzyme that displays evidence of allosteric regulation by its substrate (Piotrowski et al., 2003). Sekula et al. report on the crystal structures of wild type and mutant CPA enzymes from *Medicago truncatula*. They show that this protein forms an octameric structure that may provide a protective configuration allowing continued enzyme activity under stress. They also identify a secondary substrate-binding site in proximity of a more deeply embedded catalytic pocket, which may allow increased enzyme activity in response to increased substrate levels without reliance on transcriptional and/or translational regulation during stress or nodulation.

Two other papers published in this Topical issue focused on another key enzyme in the biosynthesis of putrescine-derived polyamines: S-adenosylmethionine decarboxylase (SAMDC). Mellidou et al. show that tobacco plants transformed with anti-sense constructs aimed at down-regulating SAMDC displayed reduced vigor under salt stress conditions while demonstrating increased biomass production relative to wild type under control conditions. Salt sensitivity of these plants seemed to derive from the inability to control H_2_O_2_ levels as well as monovalent and divalent cation concentrations, suggesting that polyamines may regulate the trade-off between growth and tolerance response. In a related paper, Goyal et al. demonstrate that tomato plants engineered to express yeast SAMDC accumulate more spermidine and spermine than wild type in their fruits upon exposure to long-lasting low temperature and re-warming. A 14 kD pathogenesis-related PR1b1 protein also accumulated in fruits under these conditions. The authors propose that polyamine-mediated sustained accumulation of PR1b1 in post-warmed chilled tomato fruits is a pre-emptive cold-stress response potentially contributing to cold stress-induced disease resistance.

An essential role of spermidine in eukaryotes is to provide the 4-aminobutyl moiety required for the conversion of a conserved lysine in the translation elongation factor eIF5A to the unusual amino acid hypusine, leading to factor activation. By using a conditional RNAi approach, Belda-Palazón et al. revealed that knock-down of a gene that encodes *Arabidopsis* deoxyhypusine synthase, an enzyme that catalyzes the formation of deoxyhypusine in this pathway, affects several aspects of plant growth and development, and also confers increased sensitivity to stress conditions such as high salt, glucose, or exogenous ABA. They suggest novel functions for the spermidine-dependent hypusination pathway in plant development and responses to hormonal, nutritional and environmental signals.

Pollen development is a step during a plant's life cycle that is targeted by polyamines. Aloisi et al. summarize our understanding of the occurrence, metabolism and function of polyamines and polyamine-derived molecules throughout pollen development. They discuss critical roles played by polyamines and their conjugates in regulating transcription, structuring pollen cell wall, modulating cytoskeleton dynamics, and affecting the levels of reactive oxygen species (ROS). Hence, polyamines appear to modulate many important characteristics of pollen development, from microsporogenesis to pollen-pistil interaction during fertilization and self-incompatibility reactions.

Putrescine-derived polyamines also contribute to the unfolded protein response (UPR), a cellular stress response associated with the accumulation of unfolded proteins in the endoplasmic reticulum. Sagor et al. show that spermine serves as an inducer of the UPR pathway. They report that spermine modulates the expression and splicing of bZIP-type transcription factors associated with the UPR, a process that requires calcium-influx into the cytoplasm, mitogen-activated protein kinase kinase 9 (MKK9), mitogen-activated protein kinase 3 (MPK3) and MPK6.

The mode of polyamine transport within and between tissues has been recognized as an important contributor to polyamine function in plants. A study of polyamine-resistant mutants of Arabidopsis by Tong et al. revealed that mutations in a mesophyll-specific nitrate transporter gene affect polyamine uptake. The authors conclude that polyamine transport or metabolism is associated with nitrate transport in the parenchymal tissue of the shoot.

Polyamines are covalently conjugated to specific proteins by transglutaminases (TGases). In plants, TGases are implicated in the photosynthetic process. Ioannidis et al. show that overexpression of the plastidial TGase from maize results in an extended stroma thylakoid network and higher threshold of photoprotection activation in Arabidopsis. While TGase overexpression decreases a photoprotective “energization” quenching, qE, exogenous putrescine increases it. The emerging role of TGase and polyamine network in chloroplasts is now getting clearer.

Growing evidence suggests a key role for polyamine catabolism, as catalyzed by copper amine oxidases (CuAO) and flavin dependent polyamine oxidases (PAO), in several aspects of plant development. Tavladoraki et al. review the latest findings on the function of these catabolic enzymes, evidencing an extraordinary complexity in *CuAO* and *PAO* gene families in regard to catalytic activity, subcellular localization, expression pattern, and physiological roles of the encoded proteins. The authors also emphasize the role of both intracellular and extracellular amine oxidases in ROS production and polyamine homeostasis. Similarly, Sagor et al. report that Arabidopsis mutants with defects in cytoplasmic polyamine oxidases show increased tolerance to salinity, a process associated with reduced production of ROS and strong induction of a subset of stress-responsive genes.

Two papers discuss the roles of polyamine catabolism in the regulation of senescence in plants. Sobieszczuk-Nowicka et al. describe the involvement of polyamines in dark-induced leaf senescence in barley. They show an initial accumulation of polyamines at the beginning of senescence followed by a subsequent decrease at later phases. Their analyses suggest that putrescine supports senescence through GABA production whereas spermidine/spermine support senescence-dependent degradation processes. Similarly, Sequera-Mutiozabal et al. investigate the role of an Arabidopsis back-conversion polyamine oxidase (PAO4) in dark-induced senescence. They found that two independent loss-of-function *pao4* mutants show delayed entry into senescence under dark conditions. Using metabolomic approaches, they demonstrate accumulation of metabolites involved in redox regulation, central metabolism, and signaling in the mutants relative to wild type. The data support a priming status against oxidative stress. The authors hypothesize the occurrence of metabolic interactions between polyamines, particularly spermine, with cell oxidative balance and transport/biosynthesis of amino acids as a strategy to cope with oxidative damage produced during senescence.

Atanasov et al. take advantage of the natural variation existing between Arabidopsis accessions to elucidate the effect of guazatine, an antifungal compound shown to inhibit polyamine oxidases, on plant growth. A locus is identified on chromosome one as associated with guazatine tolerance, and *CHLOROPHYLLASE 1 (CHL1)* is identified as a candidate gene within this locus. Using reverse genetics, the authors identified loss-of-function mutations in *CLH1* and its paralog *CLH2*. Single and double mutants are reported to display guazatine tolerance. However, mutant plants also show wild-type polyamine profiles independently of whether they were treated with guazatine or not. The authors suggest that guazatine toxicity is overcome in some Arabidopsis accessions by mechanisms that are distinct from guazatine's activity as a polyamine oxidase inhibitor. Rather, tolerance may be associated with other mechanisms involving oxidative stress and/or membrane protection.

Unlike putrescine and its derived polyamines, cadaverine is synthesized by decarboxylation of lysine. This diamine has been implicated in plant responses to both biotic and abiotic stresses. Unfortunately, little is known about its mode of action in plants. Jancewicz et al. review the state of our knowledge on the molecular mechanisms that mediate cadaverine anabolism, catabolism, conjugation, transport, and function in plants. They suggest an important contribution to plant responses to biotic and abiotic stimuli, along with a possible signaling role between the plant and its associated microbial community.

Taken together, we believe the papers published in this Topical issue expand our understanding of the role played by polyamines in the control of plant growth, development, and response to environmental stresses. We would like to thank the many authors that contributed their insights to this topic, discussed new discoveries in the field and identified novel unresolved questions in this exciting area of research. A majority of the papers published in this Topic were authored by national and/or international collaborating teams, suggesting strong synergy within the field. Importantly, while this research topic summarizes current progress in plant polyamine biology, many unanswered questions have been identified by individual contributions, regarding *the mode of action* of polyamines and their metabolites in plant growth, development, and environmental responses, at the molecular and cellular level. We hope these unanswered questions will be viewed as exciting new areas of research in plant biology, and this topic will serve as an inspiration for further progress in this field.

## Author contributions

PM initiated this research topic. For the Editorial, all authors reviewed all Research Topic articles. PM summarized the reviews and drafted the first manuscript. RA and TT revised and modified it to get the final version. All authors approved it for publication.

### Conflict of interest statement

The authors declare that the research was conducted in the absence of any commercial or financial relationships that could be construed as a potential conflict of interest.
